# High-sensitivity C-reactive protein in heart failure with preserved ejection fraction

**DOI:** 10.1371/journal.pone.0201836

**Published:** 2018-08-16

**Authors:** Hilary M. DuBrock, Omar F. AbouEzzeddine, Margaret M. Redfield

**Affiliations:** 1 Department of Medicine, Mayo Clinic, Rochester, MN, United States of America; 2 Department of Cardiovascular Diseases, Mayo Clinic, Rochester, MN, United States of America; Kurume University School of Medicine, JAPAN

## Abstract

**Background:**

Microvascular inflammation may contribute to the pathogenesis of both heart failure with preserved ejection fraction (HFpEF) and pulmonary hypertension (PH). We investigated whether the inflammation biomarker C-reactive protein (CRP) was associated with clinical characteristics, disease severity or PH in HFpEF.

**Methods:**

Patients in the Phosphodiesterase-5 Inhibition to Improve Clinical Status and Exercise Capacity in Diastolic Heart failure (RELAX) trial had baseline high-sensitivity CRP levels measured (n = 214). Clinical characteristics, exercise performance, echocardiographic variables and biomarkers of neurohumoral activation, fibrosis and myocardial necrosis were assessed. Patients with normal (≤3mg/L) versus high (>3mg/L) CRP levels were compared.

**Results:**

The median CRP level was 3.69mg/L. CRP was elevated in 57% of patients. High CRP levels were associated with younger age, higher body mass index (BMI), chronic obstructive pulmonary disease (COPD), lower peak oxygen consumption and higher endothelin-1 and aldosterone levels. CRP increased progressively with the number of comorbidities (0.7mg/L per increment in comorbidity number, *P* = 0.02). Adjusting for age, BMI and statin use, high CRP levels were additionally associated with atrial fibrillation, right ventricular dysfunction, and higher N-terminal pro-B-type natriuretic peptide levels (*P*<0.05 for all). CRP was not associated with PH or left ventricular function. CRP did not identify responders to sildenafil(*P*-value for interaction 0.13).

**Conclusions:**

In HFpEF, high CRP is associated with greater comorbidity burden and some markers of disease severity but CRP was normal in 40% of patients. These findings support the presence of comorbidity-driven systemic inflammation in HFpEF but also the need to study other biomarkers which may better reflect the presence of systemic inflammation.

## Introduction

Comorbidity-driven systemic microvascular inflammation is postulated to play a key role in the pathogenesis of myocardial structural and functional changes in heart failure (HF) with preserved ejection fraction (HFpEF)[1]. Obesity is a prominent risk factor for HFpEF and activation of interleukin (IL)-1 and downstream IL-6 in adipose tissue is well documented [2, 3]. Epicardial coronary artery disease (CAD) is also common in HFpEF and characterized by IL-1 producing inflammatory lesions which contribute to the development and progression of atherosclerotic plaque[4, 5].

CRP is a surrogate biomarker of upstream IL-1B signaling[5]. In obesity, elevated CRP levels are associated with more severe metabolic derangements[3]. In CAD, higher CRP levels are associated with adverse vascular outcomes[5] but also future HF hospitalizations[6] and higher left ventricular filling pressures[7]. In CAD, CRP levels have been used to target anti-IL anti-inflammatory therapies[5, 8]. The impact of the IL-1 antagonist canakinumab on CRP levels was recently shown to be predictive of the long term benefit of canakinumab on major adverse cardiovascular events[9]. In HFpEF, CRP was predictive of mortality[10] and a small study demonstrated increased exercise tolerance with anti-IL therapy in patients with elevated CRP[11]. However, the prevalence of elevated CRP in HFpEF and its association with clinical characteristics and disease severity have not been well studied.

Inflammation is also postulated to contribute to the pathophysiology of pulmonary arterial hypertension (PAH; Group 1 PH)[12]. In PAH, elevated CRP levels were associated with disease severity and worse prognosis[13] and agents modulating the IL-1 pathway are being tested in PAH[12]. PH is common in HFpEF (Group 2 PH), characterized by pulmonary arterial and venous remodeling and associated with right ventricular dysfunction and worse outcomes[14–16] However, little is known regarding the relationship between CRP and Group 2 PH in HFpEF. Accordingly, we sought to determine the prevalence of elevated CRP in HFpEF and its association with clinical characteristics, PH and measures of HF severity in the well phenotyped cohort of HFpEF patients who participated in the Phosphodiesterase-5 Inhibition to Improve Clinical Status and Exercise Capacity in Diastolic Heart failure (RELAX) trial.

## Materials and methods

### Study design and study sample

The RELAX trial examined the effect of sildenafil on exercise capacity in 216 patients with HFpEF[17, 18]. The trial was conducted by the Heart Failure Clinical Research Network (HFN) and funded by the National Heart, Lung and Blood Institute (NHLBI). This manuscript was prepared using HFN RELAX research materials obtained from the NHLBI biologic specimens and data repository information coordinating center and does not necessarily reflect the opinions or views of the HFN RELAX investigators or NHLBI. The RELAX protocol was approved by the institutional review board at each participating site, and all participants provided written informed consent. Data was obtained from NHLBI and all data was fully anonymized prior to the authors’ access for this study. The Mayo Clinic Institutional Review Board waived the requirement for informed consent for this analysis.

The study design, entry criteria and results of the RELAX trial have been reported previously[17, 18]. The RELAX trial enrolled 216 outpatients with an ejection fraction ≥50% and objective evidence of HF, defined as a previous heart failure hospitalization, use of intravenous diuretic therapy for acute heart failure, use of chronic loop diuretic therapy for heart failure with left atrial enlargement, or invasively documented elevation in left ventricular filling pressures (mean pulmonary capillary wedge pressure (PCWP) >15 mmHg or left ventricular end diastolic pressure >18 mmHg). Participants were required to have reduced exercise capacity (≤60% age, sex, and body size predicted peak oxygen consumption) and an elevated N-Terminal pro-B-type natriuretic peptide (NT-proBNP) ≥400pg/ml or an elevated PCWP >20 mmHg at rest or >25 mmHg with exercise at the time of an NT-proBNP measurement of <400pg/mL). Patients with an estimated glomerular filtration rate (GFR) <20ml/min/1.73m^2^ were not eligible to participate in RELAX.

### Study tests

Participants underwent baseline studies at enrollment. These included a history and physical examination, echocardiography, cardiac magnetic resonance imaging (CMRI) if in sinus rhythm, cardiopulmonary exercise test (CPXT), 6-minute walk test, Minnesota Living with Heart Failure Questionnaire and phlebotomy for biomarker measurements. The presence of comorbidities, including hypertension, ischemic heart disease, atrial fibrillation, diabetes mellitus, and COPD were assessed by history. Obesity was defined as a BMI>30. Anemia was defined as a hemoglobin <12g/dL for women and <13g/dL for men. Chronic kidney disease was defined as a glomerular filtration rate <60 ml/min/1.73m^2^.

Comprehensive Doppler echocardiography and CMRI were performed according to study protocols with measurements performed at the HFN core echocardiography (Mayo Clinic, Rochester, Minnesota) and cardiac magnetic resonance imaging (Duke University, Durham, North Carolina) laboratories[19]. Right ventricular (RV) function was assessed visually as indeterminate, normal, or mild, moderate, or severe dysfunction. The CPXT was performed according to a RELAX-specific protocol and analyzed and interpreted by the HFN CPXT core laboratory (Massachusetts General Hospital, Boston, MA), as reported previously[18]. Predicted maximal heart rate was defined by the Astrand formula (220-age)[20]. Chronotropic incompetence was defined as a chronotropic index (change in heart rate from rest to peak exercise/age-predicted maximal heart rate minus resting heart rate) <0.8 for patients not taking beta-blockers and <0.62 for patients taking beta-blockers[20].

Plasma biomarker measurements were performed by the HFN biomarker core laboratory (University of Vermont, Burlington, Vermont), as previously described, and included biomarkers of neurohumoral activation (NT-proBNP, aldosterone, endothelin-1), renal function (cystatin-C, creatinine, uric acid), fibrosis [pro-collagen III N-terminal peptide (PIIINP), C-telopeptide for type I collagen (CITP)], myocardial injury (high-sensitivity troponin-I) and inflammation (CRP)[18]. High-sensitivity CRP (subsequently referred to as CRP throughout manuscript) was measured using the BNII nephelometer from Siemens Healthcare utilizing a particle enhanced immunonepholometric assay. Polystyrene particles were coated with monoclonal antibodies to CRP, which, in the presence of antigen (CRP), agglutinate to cause an increase in the intensity of scattered light. The increase in scattered light is proportional to the amount of CRP in the sample. The assay range is 0.16 – 1100ug/mL. Expected values for CRP in normal, healthy individuals (low to average cardiovascular risk)[21] are ≤3 mg/L. Intra-assay coefficients of variation (CV) range from 2.3–4.4% and inter-assay CVs range from 2.1–5.7%.

### Statistical analysis

Data are presented as median [interquartile ranges (IQR)] or number (percentage) for patients with normal (≤3mg/L) versus high (>3mg/L) CRP levels. Differences between groups were tested using Wilcoxon rank-sum, X^2^, Cochran-Armitage trend test, or Fisher exact tests, as appropriate. Differences within individuals between enrollment and 24 weeks were assessed using a paired t-test. Multivariable least squares linear regression was performed to assess the relationship between variables of interest and CRP levels as a dichotomous variable after adjusting for age, BMI and statin use. These variables were chosen given their previously reported association with CRP levels[22, 23]. Bi-variable and multivariable least squares linear regression was performed to assess the relationship between the number of comorbidities present at enrollment and CRP levels as a continuous variable without and with adjustment for age and statin use. The associations between CRP (log transformed) and other biomarkers (log transformed NT-proBNP, aldosterone, endothelin-1, PIIINP, CITP and hs-troponin) are presented with Pearson correlation coefficients and were tested using linear regression models without and with adjustment for age, BMI and statin use. Linear regression models adjusting for treatment group, baseline peak oxygen consumption and CRP both as a dichotomous (elevated or not) and continuous variable were used to examine the interaction between baseline CRP and treatment group on the RELAX trial primary endpoint of change in peak oxygen consumption from baseline to 24 weeks. The range (minimum-maximum) of values for variables and parameter estimates with 95% confidence intervals for unadjusted and adjusted bivariate and multivariate linear regression models are reported in the supplemental materials (Tables A-D in S1 File). Analyses were performed in SAS version 9.4 (SAS Institute, Cary, North Carolina). A two-sided *P*<0.05 was considered statistically significant.

## Results

**Fig 1:**
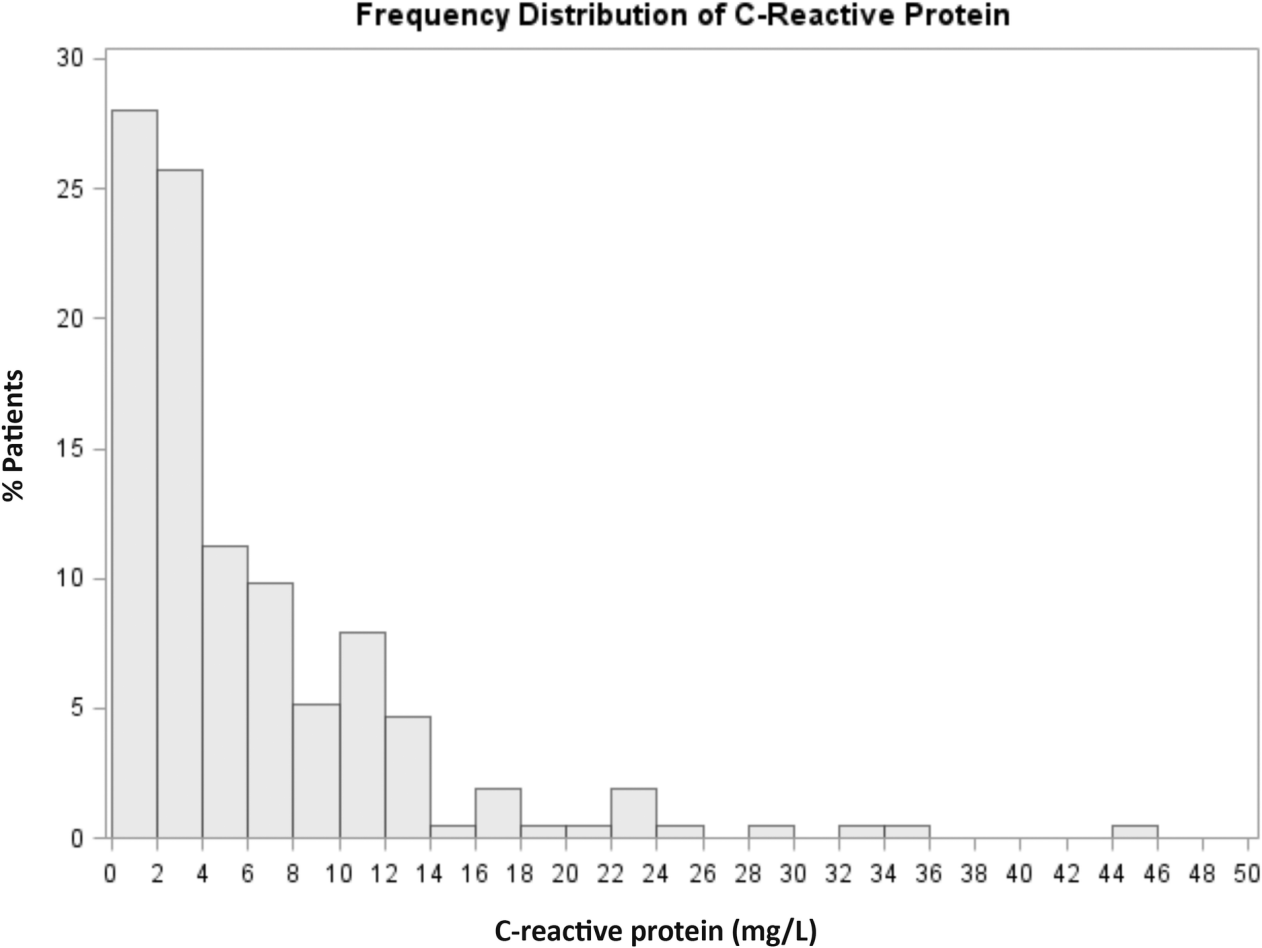
Frequency distribution of high-sensitivity C-reactive protein (CRP) levels in heart failure with preserved ejection fraction. Overall, median CRP levels were 3.69mg/L (interquartile range 1.83–8.12 mg/L with a range of 0.16 to 44.0 mg/L).

Of the 216 participants enrolled in the RELAX trial, 214 had CRP levels measured at enrollment and comprise the study group (Table 1). As previously reported, patients in the RELAX trial had a median age of 69 years, 52% were male, and comorbidities (hypertension, diabetes, atrial fibrillation (AF), obesity, Stage III/IV chronic kidney disease, COPD and ischemic heart disease) were common. Indeed, most patients had more than one of these conditions (Table 2). The median CRP level was 3.69 mg/L (IQR 1.83–8.12 mg/L) with a range of 0.16 to 44.0 mg/L (Fig 1). Fifty-seven percent (n = 121) of patients with HFpEF had elevated (> 3mg/dL) CRP levels at enrollment. Of 190 patients with repeat CRP measurements at 24 weeks, there was no significant difference between CRP levels at baseline and 24 weeks (*P* = 0.47). The median change in CRP from baseline to week 24 was -0.13 (IQR -1.50–1.48). The median percent change in CRP from baseline was -4.5% (IQR -33.3–46.4).

**Table 1:** Baseline patient characteristics by C-reactive protein levels.

	Normal CRP (≤ 3 mg/L) (n = 93)	High CRP (> 3 mg/L) (n = 121)	*P* Value	*P* Value*
**CRP, mg/L**	1.63 (0.97–2.30)	7.39 (4.35–11.40)	N/A	N/A
**Age, years**	70 (65–79)	67 (61–76)	**0.02**	N/A
**Male**	43 (46.2)	60 (49.6)	0.63	0.91
**Self-reported white race**	88 (94.6)	107 (88.4)	0.11	0.23
**Body mass index, kg/m**^**2**^	32.1 (28.5–37.2)	35.1 (31.3–40.8)	**0.004**	N/A
**Body surface area, m**^**2**^	1.99 (1.82–2.16)	2.05 (1.87–2.23)	0.05	0.40
**HF Hospitalization in last year**			0.13	0.22
0	60 (64.5)	76 (62.8)		
1	28 (30.1)	29 (24.0)		
>1	5 (5.4)	16 (13.2)		
**CV Hospitalization in last year**			0.22	0.42
0	49 (52.7)	58 (47.9)		
1	34 (36.6)	40 (33.1)		
>1	10 (10.8)	23 (19.0)		
**Comorbidities**				
Hypertension	78 (83.9)	103 (85.1)	0.80	0.74
Ischemic heart disease	35 (37.6)	47 (38.8)	0.86	0.44
Atrial fibrillation	42 (45.2)	67 (55.4)	0.14	**0.03**
COPD	12 (12.9)	30 (24.8)	**0.03**	**0.047**
Diabetes Mellitus	34 (36.6)	58 (47.9)	0.10	0.30
Malignancy	3 (3.2)	5 (4.1)	0.73	0.88
Anemia (n = 213)	34 (37.0)	52 (43.0)	0.38	0.27
Number of comorbidities (n = 211)	4 (2–5)	4 (3–5)	**0.009**	**0.003**
**Medications**				
ACE inhibitor or ARB	68 (73.1)	82 (67.8)	0.40	0.40
Aldosterone antagonist	11 (11.8)	11 (9.1)	0.51	0.56
Beta-blocker	69 (74.2)	93 (76.9)	0.65	0.29
Loop diuretic	67 (72.0)	97 (80.2)	0.16	0.13
Statin	64 (68.8)	72 (59.5)	0.16	N/A
**Laboratory Data and Biomarkers**				
Aldosterone, pg/ml	172.1 (122.1–261.8)	202.9 (117.5–312.5)	**0.03**	**0.01**
CITP I, ug/l	6.2 (4.5–9.0)	6.4 (4.9–10.2)	0.37	0.37
Creatinine, mg/dl (n = 212)	1.12 (0.89–1.34)	1.11 (0.81–1.48)	0.51	0.82
Cystatin-C, mg/l	1.25 (1.07–1.73)	1.33 (1.08–1.74)	0.77	0.43
Endothelin-1, pg/ml	2.29 (1.87–3.02)	2.44 (2.08–3.37)	**0.008**	**0.007**
GFR,ml/min/1.73m^2^ (n = 212)	61.9 (49.3–74.4)	67.1 (44.7–86.1)	0.41	0.42
Hemoglobin, mg/dl (n = 213	13.2 (12.1–14.0)	12.7 (11.9–13.5)	0.11	0.09
Hs-Troponin I, pg/ml (n = 212)	9.3 (4.8–18.3)	9.8 (5.6–19.8)	0.99	0.53
Uric acid, mg/dl (n = 212)	7.2 (5.8–8.4)	7.6 (5.9–9.0)	0.10	0.07
NT-proBNP, pg/ml (n = 213)	656 (283–1528)	719 (290–1573)	0.35	**0.01**
PIIINP,ug/l	8.1 (6.1–9.2)	7.2 (6.0–10.8)	0.78	0.74
**Congestion and Quality of Life**				
NYHA III+	45 (48.4)	69 (57.0)	0.21	0.48
MLHFQ score (n = 206)	40 (28–57)	47 (31–66)	0.08	0.36
JVP elevation (n = 207)	38 (42.2)	56 (47.9)	0.42	0.32
Moderate or severe edema	14 (15.1)	30 (24.8)	0.08	0.18
2+ pillow orthopnea (n = 211)	36 (39.1)	47 (39.5)	0.96	0.40

Abbreviations: ACE: Angiotensin converting enzyme, ARB: Angiotensin receptor blocker, CITP: C-telopeptide for type I collagen, COPD: Chronic obstructive pulmonary disease, CRP: C-reactive protein, CV: Cardiovascular, HF: Heart failure, Hs: High-sensitivity, JVP: Jugular venous pressure, MLHFQ: Minnesota Living with Heart Failure Questionnaire, NT-proBNP: N-terminal pro-B-type natriuretic peptide, NYHA: New York Heart Association, PIIINP: Pro-collagen III N-terminal peptide.

*Adjusted for age, BMI and statin use

**Table 2:** Comorbidity burden and C-reactive protein (CRP) levels. CRP levels increased as the number of comorbidities increased (parameter estimate 0.70 per increment in comorbidity number, 95%CI 0.14–1.27, *P* = 0.02), even after adjustment for age and statin use (parameter estimate 0.92, 95% CI 0.35–1.49, *P* = 0.002). Data presented as n (percent) and median (interquartile range). Comorbidities include obesity (body mass index > 30), hypertension, ischemic heart disease, atrial fibrillation, diabetes mellitus, chronic obstructive pulmonary disease, anemia, and chronic kidney disease.

Comorbidity Burden (Number of Comorbidities)	N (percent)	C-reactive protein (mg/L)
0	1 (0.5)	0.53 (0.53–0.53)
1	14 (6.6)	2.35 (1.87–8.12)
2	30 (14.2)	2.84 (1.57–6.75)
3	33 (15.6)	3.48 (1.83–6.96)
4	50 (23.7)	3.56 (2.09–8.45)
5	56 (26.5)	4.47 (1.66–8.56)
6	19 (9.0)	4.87 (2.90–11.80)
7	8 (3.8)	7.45 (4.23–23.60)

### Clinical characteristics and CRP levels in HFpEF

Participants with high CRP levels were younger (*P* = 0.02), more obese (*P* = 0.004) and were more likely to have COPD (*P* = 0.03) (Table 1). Age was inversely associated with BMI (R = -0.43, *P*<0.001). Adjusting for age, BMI and statin use, patients with high CRP levels were more likely to have AF and COPD (Table 1). There were no significant associations between CRP levels and sex, race, hospitalizations over the last year, other comorbidities, or statin or heart failure medication use in either unadjusted or adjusted analyses. Multi-morbidity was common (Table 2) and patients with elevated CRP levels had more comorbidities than those with normal CRP (Table 1). Indeed, a higher comorbidity burden was associated with higher CRP levels (Table 2) (parameter estimate 0.70 per increment in number of comorbidities, 95%CI 0.14–1.27, *P* = 0.02), even after adjustment for statin use (parameter estimate 0.83, 95% CI 0.26–1.41, *P* = 0.005).

### CRP levels and other biomarkers in HFpEF

**Fig 2:**
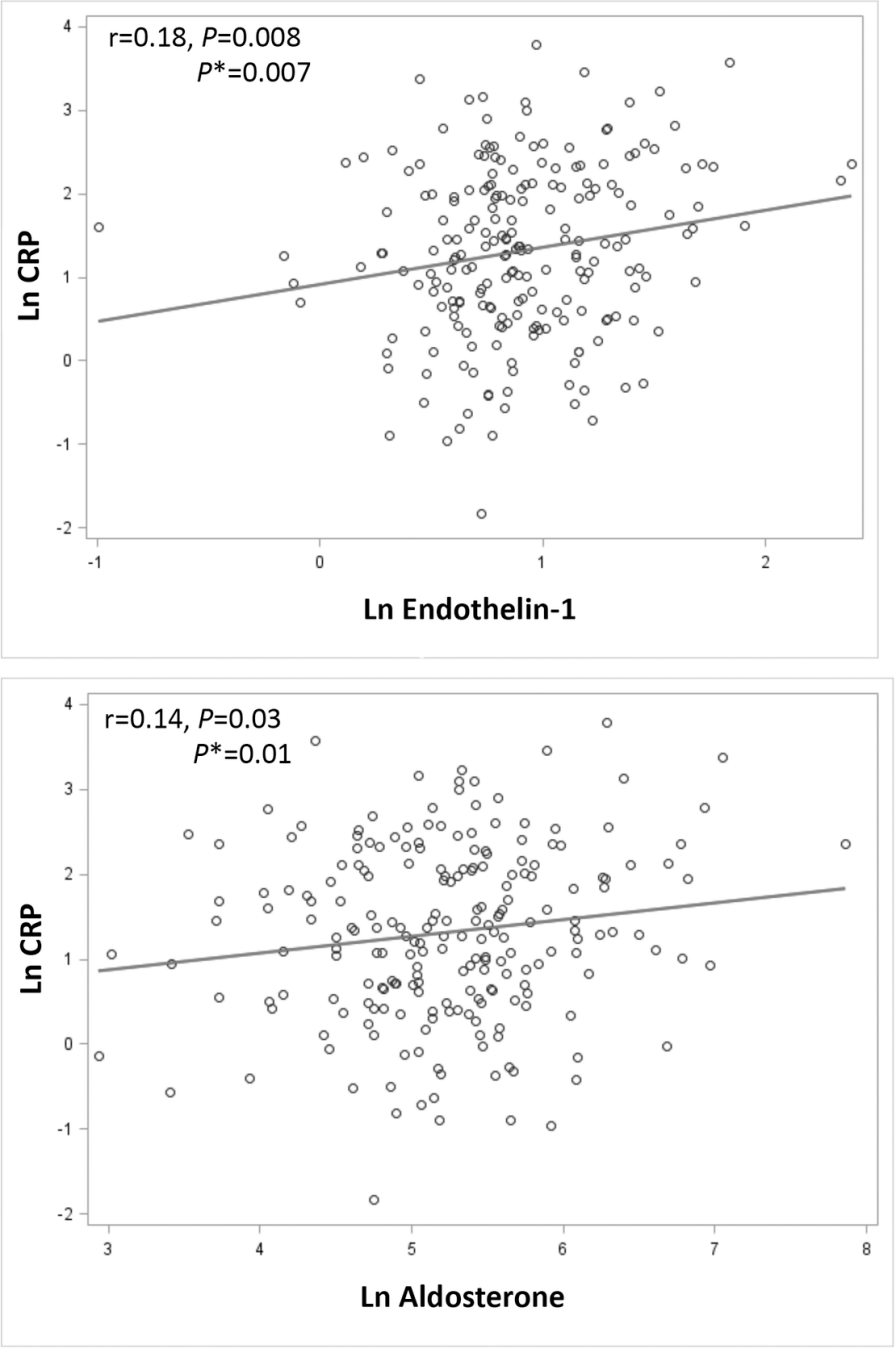
The relationship between C-reactive protein (CRP) and endothelin-1 and aldosterone in heart failure with preserved ejection fraction. CRP was significantly associated with endothelin-1 and aldosterone. Ln indicates log transformed. *Adjusted for age, BMI and statin use.

In the RELAX cohort, previous studies have reported an association between CRP and the inflammation biomarker ST-2 but not galectin-3[24, 25]. In bivariate and multivariate analysis, there were no significant associations between CRP and the fibrosis biomarkers CITP and PIIINP (r<0.10; *P*>0.05). In bivariate analysis, there were no significant associations between CRP and NT-proBNP or Troponin I, biomarkers with established prognostic value in HFpEF[26–28], although a significant association with NT-proBNP was seen after adjustment for age, BMI and statin use (parameter estimate 0.14, 95%CI 0.03–0.25, *P* = 0.01). Interestingly, in both bivariate and multivariate analysis, CRP was associated with higher levels of endothelin-1 and aldosterone (Fig 2).

### Symptoms and congestion and CRP in HFpEF

In both unadjusted and adjusted analyses, CRP was not significantly associated with heart failure symptom severity as assessed by the New York Heart Association (NYHA) functional class or Minnesota Living with Heart Failure Questionnaire score. CRP levels were not significantly associated with symptoms (orthopnea or peripheral edema) or signs (jugular venous pressure elevation) of congestion (Table 1).

### Exercise performance and CRP in HFpEF

Patients with an elevated CRP had a lower peak oxygen consumption on CPXT (*P* = 0.049) with similar effort, as reflected by the respiratory exchange ratio (*P =* 0.34)(Table 3). This relationship persisted after adjustment for age, BMI and statin use (*P* = 0.02). There were no significant differences between the groups in other parameters, peak systolic blood pressure, resting or peak heart rate, chronotropic incompetence, 6-minute walk distance, peak Borg dyspnea score, and peak oxygen saturation (Table 3). Changes in CRP from enrollment to week 24 did not correlate with changes in peak oxygen consumption (R = -0.07, *P* = 0.32).

**Table 3:** Exercise performance by baseline C-reactive protein levels (CRP).

	Normal CRP (≤3mg/L)	High CRP (>3mg/L)	*P* Value	*P* Value*
Peak VO_2_, ml/kg/minute (n = 213)	12.1 (10.5–15.1)	11.5 (10.0–13.7)	**0.049**	**0.02**
Peak Respiratory exchange ratio (n = 213)	1.08 (1.02–1.15)	1.10 (1.03–1.17)	0.34	0.05
Peak systolic BP, mmHg (n = 207)	158 (140–172)	148 (128–169)	0.11	0.11
Rest HR, beats/min (n = 211)	66 (60–76)	70 (62–79)	0.07	0.31
Peak HR, beats/min (n = 211)	107 (89–123)	111 (93–130)	0.19	0.81
Chronotropic incompetence (n = 211)	72 (78.3)	92 (77.3)	0.87	0.85
Peak VE (n = 212)	46 (34–56)	45 (35–57)	0.94	0.37
6-min walk distance, m	312 (253–396)	305 (200–368)	0.14	0.14
Peak Borg Dyspnea (n = 195)	7 (5–9)	7 (5–9)	0.69	0.35
Peak Oxygen saturation (n = 197)	96 (94–98)	96 (93–98)	0.20	0.24
Watts (n = 212)	72 (54–91)	71 (50–88)	0.51	0.14

Abbreviations: BP: Blood pressure, HR: Heart rate, VE: Minute ventilation, VO_2_: Oxygen consumption.

*Adjusted for age, BMI and statin use.

### Cardiac function and CRP in HFpEF

There was no significant association between CRP levels and diastolic function, left ventricular systolic function and geometry, or vascular function (Table 4). In unadjusted analysis, there were no significant associations with pulmonary artery systolic pressure (PASP), estimated right atrial pressure, or the presence of RV dysfunction (mild, moderate or severe). After adjustment for age, BMI and statin use, patients with higher CRP levels were more likely to have RV dysfunction (23.5% vs. 13.5%, *P* = 0.01) but there was no difference in PASP between the groups (*P* = 0.55) (Table 4).

**Table 4:** Baseline cardiac function by C-reactive protein levels.

	Normal CRP (≤3mg/L)	High CRP (>3mg/L)	*P* Value	*P* Value*
**Diastolic function parameters**				
E/A ratio (n = 140)	1.38 (0.91–3.00)	1.50 (1.00–2.00)	0.58	0.12
Medial e’, m/s (n = 195)	0.06 (0.04–0.07)	0.06 (0.05–0.08)	0.24	0.57
Medial E/e’ (n = 186)	15.0 (11.1–25.0)	16.7 (12.2–22.0)	0.76	0.54
Deceleration time, ms (n = 191)	190 (158–221)	181 (151–212)	0.20	0.35
LA volume/BSA, ml/m^2^ (n = 148)	46.1 (38.1–61.2)	45.1 (36.1–62.2)	0.90	0.43
**LV systolic function and geometry**				
Ejection fraction, %	60 (55–65)	60 (55–66)	0.34	0.51
LVEDd/BSA, cm/m^2^ (n = 162)	2.29 (2.16–2.48)	2.29 (2.10–2.52)	0.87	0.60
LV mass/BSA, g/m^2^ (n = 116)	65.4 (55.5–77.3)	65.7 (56.3–83.4)	0.68	0.73
**RV load and function**				
RA pressure, mm Hg (n = 212)	5 (5–10)	5 (5–10)	0.72	0.61
PASP, mm Hg (n = 136)	41 (32–48)	43 (34–51)	0.30	0.55
RV dysfunction (n = 204)	12 (13.5)	27 (23.5)	0.07	**0.01**
More than trivial TR (n = 198)	51 (58.6)	62 (55.9)	0.70	0.57
**Vascular function**				
Systolic BP, mm Hg	126 (112–140)	126 (114–137)	0.60	0.46
Diastolic BP, mm Hg	70 (62–80)	70 (63–78)	0.56	0.23
Ao distensibility, 10^−3^ mm Hg^-1^ (n = 86)	1.12 (0.57–1.70)	1.20 (0.73–2.25)	0.32	0.36

Abbreviations: Ao: aortic, BP: blood pressure, BSA: body surface rea, LA:left atrial, LV: left ventricular, LVEDd: left ventricular end-diastolic dimension, PASP: Pulmonary artery systolic pressure, RA: right atrial, RV: Right ventricular, TR: Tricuspid regurgitation.

*Adjusted for age, BMI and statin use

### CRP as a biomarker of response to phosphodiesterase-5 inhibition

There was no significant interaction between baseline CRP levels and treatment group (sildenafil vs. placebo) on the RELAX trial primary endpoint of change in peak oxygen consumption after 6 months of therapy (*P*-value for interaction 0.13 for CRP as a dichotomous variable and 0.07 for CRP as a continuous variable).

## Discussion

In this well-characterized cohort of patients with relatively advanced HFpEF, 57% of patients had high circulating CRP levels, and elevated CRP was associated with younger age, obesity, a diagnosis of COPD, decreased peak oxygen consumption and greater neurohumoral activation (aldosterone and endothelin-1). After adjusting for age, BMI and statin use, patients with high CRP had decreased peak oxygen consumption, higher levels of aldosterone, endothelin-1 and NT-proBNP, and were more likely to have COPD, AF, and RV dysfunction. CRP was not associated with sex, race, other comorbidities, such as diabetes mellitus and ischemic heart disease, symptoms or signs of congestion, biomarkers of myocardial fibrosis or necrosis, or echocardiographic parameters of RV load (PASP) or LV diastolic or systolic function. Importantly, CRP levels increased in proportion to the number of pro-inflammatory comorbidities present in each patient. These data are pertinent to the proposed comorbidity-driven microvascular inflammation pathologic paradigm for HFpEF and to consideration of inflammation as a therapeutic target in HFpEF[1].

### CRP in HFpEF

Overall, CRP was elevated in approximately 60% of patients with HFpEF. Median CRP in the RELAX cohort (3.69 mg/L) was similar to prior studies in patients with HFpEF in which the median CRP was between 3 and 4mg/L[7, 10, 29]. A large registry of patients referred for coronary angiography identified 459 patients with HFpEF based on hemodynamic or BNP assay criteria. In this registry, CRP was associated with several pro-inflammatory comorbidities and markers of HF severity (BNP and NYHA class) and was predictive of all-cause and cardiovascular mortality, even in patients without CAD[10]. Other studies have found that CRP in combination with other biomarkers of inflammation and neurohumoral activation, such as ST-2, Growth differentiation factor-15, and NT-pro BNP may be useful in prognostication in HF, although these studies had relatively few patients with HFpEF[30, 31].

Given the high burden of pro-inflammatory comorbidities known to be associated with higher CRP levels in the RELAX HFpEF cohort and the fairly advanced nature of the HF in the RELAX cohort, it is perhaps surprising that 40% of HFpEF patients did not have elevated CRP. It is not known whether these patients with normal CRP levels represent a distinct phenotype of HFpEF with different pathophysiologic mechanisms or whether circulating CRP levels are insensitive biomarkers of microvascular inflammation. Westermann et al previously described microvascular and myocardial inflammation and increased accumulation of collagen on endomyocardial biopsy specimens of patients with HFpEF[32]. Despite myocardial accumulation of inflammatory cells and fibrosis, circulating CRP levels in patients with HFpEF were not elevated and were similar to controls[32]. This study was small (20 HFpEF patients), patients were fairly young (mean age 60 years) and the clinical phenotype was not extensively described. However, this study does suggest that circulating CRP levels may not be a sensitive biomarker of cardiac microvascular and myocardial inflammation in HFpEF.

### CRP and clinical characteristics and comorbidities in HFpEF

Consistent with findings in other cohorts, CRP was inversely associated with age and directly associated with BMI[22]. Younger patients had a higher BMI on average and were more likely to be obese (BMI>30 kg/m^2^). CRP was not associated with race or sex, although patients in the RELAX cohort were predominantly white. Previous studies have reported conflicting results regarding associations between CRP and sex in HF.[7, 10, 29] These inconsistencies may be due to the nature of the base cohorts from which HFpEF patients were identified or differences in other confounding variables, such as BMI, comorbidities and background therapy. Similar to previous reports in other disease cohorts, we found that high CRP levels were associated with both AF and COPD, but we did not identify an association between CRP and other individual comorbidities, such as diabetes mellitus and ischemic heart disease[7, 33, 34]. As above, since CRP can be influenced by a variety of factors, the global burden of pro-inflammatory comorbidities may be more predictive of inflammation than any one individual condition.

### CRP and other biomarkers in HFpEF

In bivariate analysis, high CRP levels were not significantly associated with biomarkers of myocardial fibrosis or necrosis but were associated with biomarkers of neurohumoral activation (endothelin-1 and aldosterone). After adjusting for age, BMI and statin use, however, high CRP levels were associated with elevated aldosterone, endothelin-1 and to a lesser degree, NT-proBNP levels. Although the mechanism cannot be elucidated from this analysis, these findings suggest an association between neurohumoral activation and inflammation in the setting of HFpEF. These biomarkers of neurohumoral activation can be elevated in the setting of both heart failure and pulmonary hypertension and are associated with disease severity and worse prognosis[28, 35–38]. The relationship between CRP and NT-proBNP, a biomarker with reduced sensitivity for the diagnosis of HF in the setting of obesity, was only significant after adjusting for other variables, such as BMI[39]. These results underscore the importance of adjusting for confounding variables, such as BMI and statin use (known to reduce CRP levels), when assessing the associations between CRP and other parameters.

### CRP and symptoms and congestion in HFpEF

There was no significant difference between the groups in symptoms or signs of congestion, HF specific quality of life scores or hospitalizations within the last year. A large coronary angiogram HFpEF registry reported an association between CRP and worse NYHA functional class in HF[10], but we did not observe this in the RELAX cohort where patients were predominantly class II or III. This may also reflect differences in observational vs clinical trial populations or the referral bias in the angiographic registry.

### CRP and exercise performance in HFpEF

Consistent with other studies in patients with predominantly systolic HF[40, 41], we found that higher CRP levels were associated with exercise intolerance and decreased peak oxygen consumption. In a pilot cross-over study of 12 patients with HFpEF and elevated CRP, decreases in circulating CRP with 14 days of treatment with the IL-1 receptor antagonist anakinra correlated with increases in peak oxygen consumption[11]. In the RELAX cohort, CRP at enrollment was associated with lower peak oxygen consumption at enrollment, but changes in CRP from enrollment to week 24 did not correlate with changes in peak oxygen consumption. This may have been due to differences in enrollment criteria (the anakinra study only included patients with a baseline CRP>2mg/L) and larger percent changes in CRP associated with anakinra use. In the anakinra study, there was a 74% reduction in CRP whereas there was only a 4.5% change in CRP from enrollment to week 24 in the RELAX study.

### CRP and cardiac function in HFpEF

In HFpEF, we did not find a significant association between CRP and PH, as assessed by PASP, or between CRP and parameters of LV systolic or diastolic function. After adjusting for age, BMI and statin use, high CRP levels were associated with RV dysfunction, but not PASP. Similarly, in patients with PAH, CRP did not correlate with PASP, but was associated with other markers of disease severity and a worse prognosis[13]. The authors of the PAH study postulated that the worse prognosis in patients with high CRP in PAH may be due to its association with RV dysfunction. CRP as a surrogate biomarker of inflammation may reflect differences in RV adaptation to a pressure load. Microvascular or systemic inflammation may mediate differences in RV adaptation and function or CRP may simply be a downstream biomarker of systemic inflammation induced by RV dysfunction. In subjects without clinical cardiovascular disease, CRP has been associated with differences in RV morphology as assessed by MRI. The authors of the MRI study hypothesized that systemic inflammation contributes to structural changes in the RV, independent of pressure load[42]. Our finding that high CRP levels were associated with RV, but not LV, dysfunction also suggests that systemic or microvascular inflammation may have distinct effects on RV structure and function. In addition to differences we observed in RV dysfunction, we also found significant associations between CRP and NT-proBNP, an important prognostic indicator and biomarker of RV structure and function, and endothelin-1, a biomarker of both PAH and PH associated with left sided heart disease[35, 36, 43, 44]. These findings reinforce the association we observed between CRP and RV dysfunction.

### CRP as a predictor of treatment response to sildenafil in HFpEF

We hypothesized that high CRP levels would be associated with more advanced PH and therefore may identify a group of patients more likely to benefit from sildenafil use. Since PH associated with HFpEF is a heterogeneous disease without effective treatment options, it is important to identify subgroups, or phenotypes, that may respond differentially in clinical trials. In the CORONA study, for example, a significant interaction was identified between CRP levels and treatment effect of rosuvastatin[23]. Although patients with high CRP had more RV dysfunction, we did not identify a significant interaction between CRP levels and effect of sildenafil on exercise capacity.

### Limitations

This was an exploratory analysis to assess the association between CRP and PH and other clinical correlates in HFpEF with the aim of generating hypotheses that could be tested in future studies. No correction for multiple comparisons was made and this may result in discovery of associations due to chance. The range of CRP levels at enrollment was wide, and we were unable to adjust for confounding factors, such as subclinical infection. The association between CRP levels and mortality was not examined as events were low in the 6 month RELAX study. Lastly, we were limited to the use of echocardiogram, rather than invasive hemodynamic testing, to assess PH severity in patients with HFpEF, and PASP estimates were not available on all participants and assessment of RV function was only visual and not quantified.

### Conclusions

In HFpEF, CRP levels were elevated in approximately 60% of patients and were associated with younger age, higher BMI, COPD, AF, RV dysfunction, reduced exercise tolerance and higher circulating levels of aldosterone, endothelin-1 and NT-pro BNP, but were not significantly associated with symptoms, PASP or other parameters of LV systolic or diastolic function. High CRP levels may identify a unique phenotype of HFpEF that is associated with more systemic inflammation related to the presence of multiple pro-inflammatory comorbidities. However, as a non-specific systemic biomarker of inflammation, CRP levels are influenced by numerous factors and disease states, and may not sensitively or specifically reflect cardiac microvascular inflammation in HFpEF. These data add to our understanding of CRP as a biomarker of inflammation in HFpEF, underscore the need to adjust for confounding variables when assessing the relationship between CRP and other parameters and suggest the need to study alternate biomarkers which may better reflect “comorbidity driven systemic inflammation” in HFpEF or specific HFpEF phenotypes.

## Supporting information

S1 File Supplemental tables. Supplemental tables with minimum to maximum values for all variables as well as parameter estimates with 95% confidence intervals for bivariate and multivariate linear regression models are included in the supplemental information. **Table A. Baseline patient characteristics by C-reactive protein levels.** Data reported as the range of minimum-maximum values or number (percent). Parameter estimates with 95% confidence intervals are reported for bivariate linear regression models and multivariate linear regression models adjusted for age, body mass index (BMI) and statin use. Abbreviations: ACE: Angiotensin converting enzyme, ARB: Angiotensin receptor blocker, CITP: C-telopeptide for type I collagen, COPD: Chronic obstructive pulmonary disease, CRP: C-reactive protein, CV: Cardiovascular, HF: Heart failure, Hs: High-sensitivity, JVP: Jugular venous pressure, MLHFQ: Minnesota Living with Heart Failure Questionnaire, NT-proBNP: N-terminal pro-B-type natriuretic peptide, NYHA: New York Heart Association, PIIINP: Pro-collagen III N-terminal peptide. *Adjusted for age, BMI and statin use. **Table B. Comorbidity burden and range of C-reactive protein (CRP) levels.** Data presented as number (percent) and range of minimum to maximum values. Comorbidities include obesity (body mass index > 30), hypertension, ischemic heart disease, atrial fibrillation, diabetes mellitus, chronic obstructive pulmonary disease, anemia, and chronic kidney disease. **Table C. Exercise performance by baseline C-reactive protein (CRP) levels.** Data reported as the range of minimum-maximum values or number (percent). Parameter estimates with 95% confidence intervals are reported for bivariate linear regression models and multivariate linear regression models adjusted for age, body mass index (BMI) and statin use. Abbreviations: BP: Blood pressure, HR: Heart rate, VE: Minute ventilation, VO_2_: Oxygen consumption *Adjusted for age, BMI and statin use. **Table D. Baseline cardiac function by C-reactive protein levels.** Data reported as the range of minimum-maximum values or number (percent). Parameter estimates with 95% confidence intervals are reported for bivariate linear regression models and multivariate linear regression models adjusted for age, body mass index (BMI) and statin use. Abbreviations: Ao: aortic, BP: blood pressure, BSA: body surface rea, LA:left atrial, LV: left ventricular, LVEDd: left ventricular end-diastolic dimension, PASP: Pulmonary artery systolic pressure, RA: right atrial, RV: Right ventricular, TR: Tricuspid regurgitation. *Adjusted for age, BMI and statin use.
(DOCX)
