# Supplementary material for: High-sensitivity C-reactive protein in heart failure with preserved ejection fraction
Source: PLoS One. 2018 Aug 16;13(8):e0201836. doi: 10.1371/journal.pone.0201836 (PMC6095520; doi:10.1371/journal.pone.0201836)
Supplement: S1 File — Supplemental tables with minimum to maximum values for all variables as well as parameter estimates with 95% confidence intervals for bivariate and multivariate linear regression models are included in the supplemental information. Table A. Baseline patient characteristics by C-reactive protein levels. Data reported as the range of minimum-maximum values or number (percent). Parameter estimates with 95% confidence intervals are reported for bivariate linear regression models and multivariate linear regression models adjusted for age, body mass index (BMI) and statin use. Abbreviations: ACE: Angiotensin converting enzyme, ARB: Angiotensin receptor blocker, CITP: C-telopeptide for type I collagen, COPD: Chronic obstructive pulmonary disease, CRP: C-reactive protein, CV: Cardiovascular, HF: Heart failure, Hs: High-sensitivity, JVP: Jugular venous pressure, MLHFQ: Minnesota Living with Heart Failure Questionnaire, NT-proBNP: N-terminal pro-B-type natriuretic peptide, NYHA: New York Heart Association, PIIINP: Pro-collagen III N-terminal peptide. *Adjusted for age, BMI and statin use. Table B. Comorbidity burden and range of C-reactive protein (CRP) levels. Data presented as number (percent) and range of minimum to maximum values. Comorbidities include obesity (body mass index > 30), hypertension, ischemic heart disease, atrial fibrillation, diabetes mellitus, chronic obstructive pulmonary disease, anemia, and chronic kidney disease. Table C. Exercise performance by baseline C-reactive protein (CRP) levels. Data reported as the range of minimum-maximum values or number (percent). Parameter estimates with 95% confidence intervals are reported for bivariate linear regression models and multivariate linear regression models adjusted for age, body mass index (BMI) and statin use. Abbreviations: BP: Blood pressure, HR: Heart rate, VE: Minute ventilation, VO2: Oxygen consumption *Adjusted for age, BMI and statin use. Table D. Baseline cardiac function by C-reactive protein leve [file pone.0201836.s001.docx]

|  | **Normal CRP**  **(≤ 3 mg/L)**  **(n=93)** | **High CRP**  **(> 3 mg/L)**  **(n=121)** | **Parameter estimate** | **95%CI** | **Parameter estimate*** | **95%CI*** |
| --- | --- | --- | --- | --- | --- | --- |
| **CRP, mg/L** | 0.16-3.00 | 3.05-44.00 | N/A | N/A | N/A | N/A |
| **Age, years**  **Male**  **Self-reported white race**  **Body mass index, kg/m^2^**  **Body surface area, m^2^** | 37-90  43 (46.2)  88 (94.6)  21.0-55.6  1.44-2.48 | 32-85  60 (49.6)  107 (88.4)  21.3-54.5  1.58-2.62 | -0.008  0.033  -0.188  0.012  0.314 | -0.014- -0.001  -0.101-0.167  -0.423-0.046  0.003-0.022  0.043-0.585 | N/A  0.008  -0.142  N/A  0.146 | N/A  -0.128-0.144  -0.374-0.092  N/A  -0.196-0.487 |
| **HF Hospitalization in last year**  **0**  **1**  **>1**  **CV Hospitalization in last year**  **0**  **1**  **>1** | 60 (64.5)  28 (30.1)  5 (5.4)  49 (52.7)  34 (36.6)  10 (10.8) | 76 (62.8)  29 (24.0)  16 (13.2)  58 (47.9)  40 (33.1)  23 (19.0) | Reference  -0.050  0.203  Reference  -0.002  0.155 | -0.204-0.104  -0.025-0.432  -0.149-0.146  -0.040-0.350 | Reference  -0.074  0.145  Reference  -0.013  0.118 | -0.227-0.078  -0.085-0.374  -0.159-0.133  -0.077-0.313 |
| **Comorbidities**  **Hypertension**  **Ischemic heart disease**  **Atrial fibrillation**  **COPD**  **Diabetes Mellitus**  **Malignancy**  **Anemia (n=213)** | 78 (83.9)  35 (37.6)  42 (45.2)  12 (12.9)  34 (36.6)  3 (3.2)  34 (37.0) | 103 (85.1)  47 (38.8)  67 (55.4)  30 (24.8)  58 (47.9)  5 (4.1)  52 (43.0) | 0.024  0.013  0.100  0.185  0.114  0.062  0.069 | -0.162-0.209  -0.125-0.151  -0.033-0.234  0.018-0.352  -0.021-0.249  -0.292-0.416  -0.075-0.198 | 0.032  0.056  0.148  0.169  0.078  0.028  0.075 | -0.157-0.222  -0.086-0.198  0.014-0.283  0.002-0.335  -0.071-0.228  -0.324-0.381  -0.060-0.210 |
| **Number of Comorbidities (n=211)** | 0-7 | 1-7 | 0.062 | 0.019-0.105 | 0.072 | 0.025-0.120 |
| **Medications**  **ACE inhibitor or ARB**  **Aldosterone antagonist**  **Beta-blocker**  **Loop diuretic**  **Statin** | 68 (73.1)  11 (11.8)  69 (74.2)  67 (72.0)  64 (68.8) | 82 (67.8)  11 (9.1)  93 (76.9)  97 (80.2)  72 (59.5) | -0.063  -0.073  0.036  0.111  -0.099 | -0.209-0.084  -0.294-0.148  -0.121-0.192  -0.046-0.269  -0.238-0.040 | -0.062  -0.064  0.086  0.122  N/A | -0.208-0.084  -0.282-0.154  -0.073-0.245  -0.037-0.282  N/A |
| **Laboratory Data and Biomarkers**  **Aldosterone, pg/ml**  **CITP I, ug/l**  **Creatinine, mg/dl (n=212)**  **Cystatin-C, mg/l**  **Endothelin-1, pg/ml**  **GFR,ml/min/1.73m^2^ (n=212)**  **Hemoglobin, mg/dl (n=213)**  **Hs-Troponin I, pg/ml (n=212)**  **Uric acid, mg/dl (n=212)**  **NT-proBNP, pg/ml (n=213)**  **PIIINP,ug/l** | 18.9-1066.9  0.3-30.4  0.67-2.65  .0.79-3.12  0.89-5.39  23.5-114.6  10.0-16.4  2.3-132.3  0.5-11.3  28-3106  3.5-21.8 | 34.1-2600.0  0.3-24.1  0.44-3.31  0.68-3.14  0.37-10.87  26.2-150.1  9.5-18.3  1.8-549.9  1.9-12.6  15-8052  2.9-50.0 | 0.197  0.098  0.002  0.038  0.444  0.002  -0.031  -0.001  0.032  0.051  -0.049 | 0.014-0.380  -0.119-0.315  -0.142-0.146  -0.091-0.167  0.118-0.770  -0.001-0.004  -0.076-0.014  -0.131-0.130  0.002-0.063  -0.055-0.156  -0.388-0.290 | 0.222  0.096  0.016  0.054  0.446  0.001  -0.038  0.041  0.028  0.143  -0.055 | 0.046-0.399  -0.116-0.308  -0.129-0.161  -0.081-0.188  0.125-0.767  -0.002-0.004  -0.083-0.006  -0.087-0.168  -0.002-0.059  0.033-0.254  -0.382-0.273 |
| **Congestion and Quality of Life**  **NYHA III+**  **MLHFQ score (n=206)**  **JVP elevation (n=207)**  **Moderate or severe edema**  **2+ pillow orthopnea (n=211)** | 45 (48.4)  4-88  38 (42.2)  14 (15.1)  36 (39.1) | 69 (57.0)  0-105  56 (47.9)  30 (24.8)  47 (39.5) | 0.085  0.003  0.056  0.147  0.004 | -0.049-0.219  -0.0003-0.006  -0.081-0.193  -0.018-0.311  -0.135-0.142 | 0.050  0.001  0.071  0.119  -0.062 | -0.088-0.188  -0.002-0.005  -0.069-0.212  -0.054-0.291  -0.205-0.082 |

**S1 Table A.**

| **Comorbidity Burden** (Number of Comorbidities) | **N (percent)** | **C-reactive protein (mg/L)**  **Minimum-Maximum** |
| --- | --- | --- |
| 0 | 1 (0.5) | 0.53-0.53 |
| 1 | 14 (6.6) | 0.41-44.0 |
| 2 | 30 (14.2) | 0.44-16.30 |
| 3 | 33 (15.6) | 0.16-13.40 |
| 4 | 50 (23.7) | 0.38-23.60 |
| 5 | 56 (26.5) | 0.49-29.10 |
| 6 | 19 (9.0) | 0.97-35.70 |
| 7 | 8 (3.8) | 1.11-32.00 |

**S1 Table B.**

|  | **Normal CRP**  **(≤3mg/L)** | **High CRP**  **(>3mg/L)** | **Parameter estimate** | **95%Confidence Interval** | **Parameter estimate*** | **95%Confidence Interval*** |
| --- | --- | --- | --- | --- | --- | --- |
| Peak VO_2_, ml/kg/minute (n=213) | 5.8-20.3 | 5.3-19.3 | -0.023 | -0.044- -0.001 | -0.029 | -0.052- -0.006 |
| Peak Respiratory exchange ratio (n=213) | 0.80-1.60 | 0.89-1.44 | 0.373 | -0.258-1.004 | 0.651 | -0.007-1.31 |
| Peak systolic BP, mmHg (n=207) | 83-246 | 74-240 | -0.001 | -0.004-0.001 | -0.002 | -0.004-0.000 |
| Rest HR, beats/min (n=211) | 45-109 | 45-101 | 0.004 | -0.001-0.009 | 0.003 | -0.003-0.008 |
| Peak HR, beats/min (n=211) | 60-187 | 64-190 | 0.001 | -0.001-0.004 | 0.0003 | -0.003-0.003 |
| Chronotropic incompetence (n=211) | 72 (78.3) | 92 (77.3) | -0.013 | -0.176-0.149 | -0.015 | -0.176-0.146 |
| Peak VE (n=212) | 17-115 | 20-84 | -0.000 | -0.005-0.004 | -0.002 | -0.007-0.003 |
| 6-min walk distance, m | 66-525 | 23-547 | -0.001 | -0.001-0.000 | -0.0005 | -0.001-0.000 |
| Peak Borg Dyspnea (n=195) | 0.5-10.0 | 1-10 | -0.008 | -0.038-0.022 | -0.014 | -0.045-0.016 |
| Peak Oxygen saturation (n=197) | 85-100 | 79-100 | -0.017 | -0.036-0.003 | -0.012 | -0.033-0.008 |
| Watts (n=212) | 5-153 | 0-165 | -0.001 | -0.003-0.001 | -0.002 | -0.004-0.001 |

**S1 Table C.**

|  | **Normal CRP**  **(≤3mg/L)** | **High CRP**  **(>3mg/L)** | **Parameter**  **estimate** | **95%Confidence Interval** | **Parameter**  **Estimate*** | **95% Confidence Interval*** |
| --- | --- | --- | --- | --- | --- | --- |
| **Diastolic function parameters**  E/A ratio (n=140)  Medial e’, m/s (n=195)  Medial E/e’ (n=186)  Deceleration time, ms (n=191)  LA volume/BSA, ml/m^2^ (n=148) | 0.50-11.00  0.02-0.11  5.5-45.0  99-317  13.6-184.8 | 0.63-4.67  0.03-0.13  6.0-60.0  99-400  23.1-132.9 | -0.056  1.825  0.001  -0.001  -0.0001 | -0.119-0.006  -1.436-5.085  -0.007-0.008  -0.002-0.001  -0.004-0.004 | -0.048  0.947  0.002  -0.001  0.002 | -0.110-0.013  -2.340-4.234  -0.005-0.010  -0.002-0.001  -0.002-0.006 |
| **LV systolic function and geometry**  Ejection fraction, %  LVEDd/BSA, cm/m^2^ (n=162)  LV mass/BSA, g/m^2^ (n=116) | 50-83  1.62-3.00  40.5-151.8 | 50-79  1.63-3.23  40.4-170.8 | 0.002  0.035  -0.0003 | -0.008-0.012  -0.219-0.289  -0.004-0.003 | 0.003  0.070  -0.001 | -0.007-0.013  -0.193-0.333  -0.004-0.003 |
| **RV load and function**  RA pressure, mm Hg (n=212)  PASP, mm Hg (n=136)  RV dysfunction (n=204)  More than trivial TR (n=198) | 5-20  23-72  12 (13.5)  51 (58.6) | 5-20  19-74  27 (23.5)  62 (55.9) | 0.004  0.003  0.159  -0.028 | -0.011-0.018  -0.004-0.010  -0.015-0.333  -0.169-0.113 | 0.004  0.002  0.224  0.045 | -0.011-0.018  -0.005-0.009  0.051-0.398  -0.113-0.203 |
| **Vascular function**  Systolic BP, mm Hg  Diastolic BP, mm Hg  Ao distensibility, 10^-3^ mm Hg^-1^ (n=86) | 101-176  48-98  0.12-4.53 | 98-177  41-94  0.20-6.70 | -0.002  -0.003  0.054 | -0.006-0.002  -0.009-0.004  -0.038-0.147 | -0.001  -0.004  0.054 | -0.005-0.002  -0.010-0.002  -0.062-0.171 |

**S1 Table D.**
